# Industry 4.0 Quantum Strategic Organizational Design Configurations. The Case of 3 Qubits: One Reports to Two

**DOI:** 10.3390/e23030374

**Published:** 2021-03-20

**Authors:** Javier Villalba-Diez, Juan Carlos Losada, Rosa María Benito, Ana González-Marcos

**Affiliations:** 1 Hochschule Heilbronn, Fakultät Management und Vertrieb, Campus Schwäbisch Hall, 74523 Schwäbisch Hall, Germany; 2 Complex Systems Group, Universidad Politécnica de Madrid, Av. Puerta de Hierro 2, 28040 Madrid, Spain; juancarlos.losada@upm.es (J.C.L.); rosamaria.benito@upm.es (R.M.B.); 3 Department of Mechanical Engineering, Universidad de La Rioja, 26004 Logroño, Spain; ana.gonzalez@unirioja.es

**Keywords:** quantum strategic organizational design, industry 4.0, quantum circuits

## Abstract

In this work we explore how the relationship between one subordinate reporting to two leaders influences the alignment of the latter with the company’s strategic objectives in an Industry 4.0 environment. We do this through the implementation of quantum circuits that represent decision networks. This is done for two cases: One in which the leaders do not communicate with each other, and one in which they do. Through the quantum simulation of strategic organizational design configurations (QSOD) through 500 quantum circuit simulations, we conclude that in the first case both leaders are not simultaneously in alignment, and in the second case that both reporting nodes need to have an alignment probability higher than 90% to support the leader node.

## 1. Introduction

According to Grant [1], strategic planning in an organization consists of the principle of the strategic process: “A dialogue through which knowledge is shared and consensus and commitment toward action and results are achieved.” In this dialogue, described above as Nemawashi [2] or “catch-ball” [3] by scholars, is where a balance of forces, sometimes delicate, is sought between the interests of different organizational agents [4]. Under a strategic organizational design paradigm [5,6], the interrelation of these interdependent organizational elements shapes complex hierarchical networks [7] and supports decision making in order to achieve, ideally, a coordination of efforts in pursuit of the organization’s strategic objectives called organizational alignment. Such alignment efforts can occur in different organizational environments, although in this paper the authors focus on complex networked cyber-physical systems in an Industry 4.0 context.

Approaches to qualitatively model organizational alignment have been proposed by several scholars [8,9,10,11,12,13,14]. Less common are approaches that allow quantifying the organizational alignment [2], where the alignment status of each node is known at each discrete time interval. The NEMAWASHI approach, based on genetic algorithms, is however computationally very expensive and therefore difficult to implement in practice. Although the computation of the alignment state of the entire network is theoretically possible with this method, in practice it is a challenge that leads to an exponential increase in computational time with increasing network size. For this reason, there is an urgent need to provide organizational leaders with a fast algorithm to calculate the alignment state of the organization.

Quantum computing is a novel computing paradigm that could be useful for this purpose. In quantum computing, information flow and processing are considered to be physical phenomena governed by the laws of quantum mechanics. It is possible because quantum computing makes use of “superposition,” that is, the ability of quantum computers to be simultaneously in multiple different states [15]. Thus, quantum computing has shown promising performance gains in solving certain problems unattainable for classical computing. Shor’s algorithm [16] and Grover’s algorithm [17] are two paradigmatic examples of quantum superior computational performance when compared to classical algorithms.

Guiding an organization toward the coordinated accomplishment of strategic objectives is a probabilistic process in which decision makers can never be sure that the choice made is the right one. Decision-makers are conditioned by the simultaneous decisions of other actors in the organization whose consequences cannot be fully foreseen a priori. Consequently, these networks can be considered decision networks or acyclic probabilistic directed graphical models [18] with known conditional probabilities of alignment. As with the aforementioned genetic algorithm approach, the implementation of this problem as a Bayesian network is computationally very expensive in the presence of a large number of nodes.

This work is conceived as a succinct outreach of an important new application of the previous work on Quantum Strategic Organizational Design (QSOD) [19,20], and should be referenced as a background by the motivated reader. The QSOD permits real-time modeling of organizational alignment conditions of complex systems in Industry 4.0. Quantum circuit simulations of QSODs as decision making networks and equivalent quantum circuits certainly open a large scope of opportunities for the study of the design of complex networked strategic organizations. As mentioned in the previous mentioned documents, in this work we depict the owner of the individual process, a complex network node in Industry 4.0 represented in the form of a decision graph [18], as a quantum computing unit or qubit [21,22]. This qubit is allowed to have two fundamental states, one of alignment or asymptotic stability of the Key Performance Indicators (KPIs) defining its performance [2,23,24,25,26,27,28,29,30,31], represented by the state |0〉 and another of non–alignment, lack of such stability, represented by the state |1〉. In the previous work [20], we showed how the interaction between two agents, an industrial leader and a subordinate reporting to him, can be interpreted as a dissipative oscillatory system in underdamped mode.

**Figure 1:**
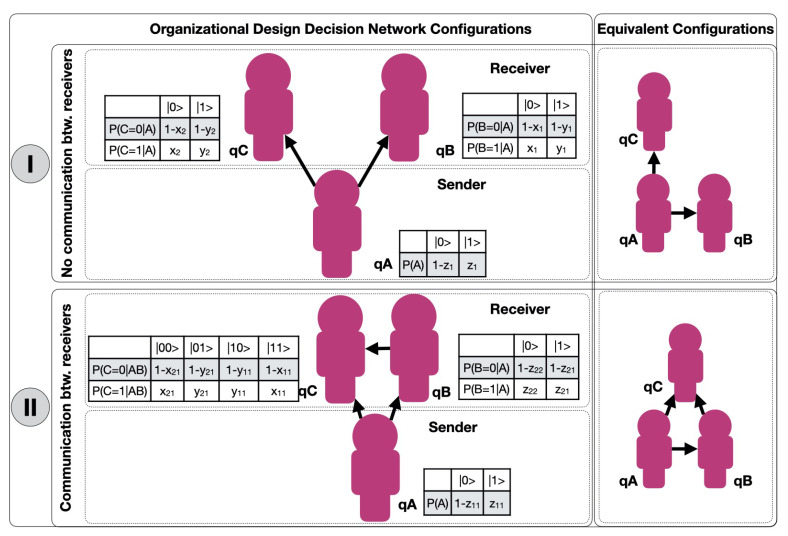
Quantum simulation of Strategic Organizational Design (QSOD). Case of three qubits configuration in which one node reports to two. I. Without communication between the leaders. II. With communication between the leaders.

As we illustrate in Figure 1, in this work we add a twist to these configurations by simulating the configuration in which one subordinate (sender) agent *A* node reports to two others (receivers) *B* and *C* in two cases: When the nodes receiving the report do not communicate with each other, and when they communicate with each other. These organizational configurations under study are indeed extremely relevant since they represent basic strategic organizational design configurations such as the relationships of hierarchically related agents (vertical relationships) or supplier–customer interactions along the value stream (horizontal relationships). In the figure we show the respective topological equivalent configurations to each case. We aim to investigate the leader’s probability of alignment, with the strategic objectives of the organization, depending on the state of the subordinates and their respective conditional probabilities of alignment between them.

The Bloch sphere is the standard qubit geometric representation [32]. The *Z*-axis of Bloch’s sphere, of unit radius, becomes the calculation axis whose positive direction coincides with the state |0〉, and the negative with the state |1〉. We can represent the state of a qubit given by |Ψ〉 by means of a point on the Bloch sphere with the help of two parameters (θ, ϕ), as expressed by Equation (1):

(1) $$|\Psi \rangle =cos\left(\frac{\theta }{2}\right)|0\rangle +e^{i\phi }sin\left(\frac{\theta }{2}\right)|1\rangle .$$

Our objective is to establish the alignment probability of agents *B* and *C*, P(B=|0〉), and P(C=|0〉) respectively, in dependence of the alignment probability of agent *A* and the conditional alignment probabilities between agents *A*, *B*, and *C*. This is accomplished by simulating hundreds of different quantum circuit configurations.

The work continues as follows: First, Section 2 begins with a description of the configuration of the quantum circuit computations necessary to simulate the outlined 3–qubit organizational design configuration. Second, Section 3 presents the two case studies describing the two presented configurations: (I) Describing the case in which agents *B* and *C* have no communication between each other, and (II) describing the case in which agents *B* and *C* have communication between each other. Throughout the simulation of numerous quantum circuits, varying the mentioned parameters, an optimal configuration of them is sought for. Third, in Section 4 we discuss the results obtained and propose an interpretation in perspective of previous studies and of the working hypotheses. Finally, in Section 5 we discuss the findings and their implications in a broad context, and future research directions and limitations are highlighted.

## 2. QSOD Circuits–3 Qubit Organizational Design Configurations–One Reports to Two

An initial hypothesis of this work is that the leader of the Industry 4.0 organization benefits from knowing its alignment status with the strategic objectives of the organization. That is why we will focus on finding answers to the question of how to maximize the probabilities of alignment of nodes *B* and *C*, P(B=|0〉), and P(C=|0〉) respectively, depending on the individual alignment probabilities of the root node *A*, as well as their respective relative probabilities between the nodes given by different parameters in the two announced cases of study.

### 2.1. Quantum Circuit–Case I–Agents B and C Have No Communication between Each Other

In this case, as shown in Figure 1I, we will represent a three-qubit system. As explained in [19,21], this requires the use of three qubits $|\Psi_{A}\rangle$, $|\Psi_{B}\rangle$, and $|\Psi_{C}\rangle$. It should be noted that since the system is symmetrical, the position of the nodes (B) and (C) are interchangeable. We are faced with a three qubit system whereby the combined state can be described as the tensor product of the individual qubits. The multiple *qubit* states can be expressed as a linear combination of the |0〉 and |1〉 states, and the aggregate state can then be represented as in Equation (2).

(2) $$\begin{array}{cc} |\Psi \rangle & =|\Psi_{A}\rangle ⊗|\Psi_{B}\rangle ⊗|\Psi_{C}\rangle \\ \; & =a_{0}b_{0}c_{0}|000\rangle +a_{0}b_{0}c_{1}|001\rangle +a_{0}b_{1}c_{0}|010\rangle +a_{0}b_{1}c_{1}|011\rangle \\ \; & +a_{1}b_{0}c_{0}|100\rangle +a_{1}b_{0}c_{1}|101\rangle +a_{1}b_{1}c_{0}|110\rangle +a_{1}b_{1}c_{1}|111\rangle \end{array}$$

where:
$|\Psi_{A}\rangle =a_{0}|0\rangle +a_{1}|1\rangle$$\;a_{i}\in C^{2}$$|\Psi_{B}\rangle =b_{0}|0\rangle +b_{1}|1\rangle$$\;b_{i}\in C^{2}$$|\Psi_{C}\rangle =c_{0}|0\rangle +c_{1}|1\rangle$$\;c_{i}\in C^{2}$.

Thus it can be said that the quantum system of 3 qubits can be described by a $2^{3}$-dimensional complex unit vector $|\Psi \rangle \in C^{2}$.

To describe the case in which agents *B* and *C* have no communication between each other we need the following parameters:

- $1-z_{1}=P\left(A=|0\rangle \right)=1-P\left(A=|1\rangle \right)$. Probability of alignment of node *A*.
- $x_{1}=P\left(B=|1\rangle |A=|0\rangle \right)$. Probability of no–alignment of node *B* conditioned to the state of alignment of node *A*.
- $y_{1}=P\left(B=|1\rangle |A=|1\rangle \right)$. Probability of no–alignment of node *B* conditioned to the state of no–alignment of node *A*.
- $x_{2}=P\left(C=|1\rangle |A=|0\rangle \right)$. Probability of no–alignment of node *C* conditioned to the state of alignment of node *A*.
- $y_{2}=P\left(C=|1\rangle |A=|1\rangle \right)$. Probability of no–alignment of node *C* conditioned to the state of no–alignment of node *A*.

Mathematically speaking, we intend to find the values of $\left(x_{1},y_{1},x_{2},y_{2},z_{1}\right)$ that maximize the functions $P\left(B=|0\rangle \right)=f_{I}\left(x_{1},y_{1},x_{2},y_{2},z_{1}\right)$ and $P\left(C=|0\rangle \right)=g_{I}\left(x_{1},y_{1},x_{2},y_{2},z_{1}\right)$. In other words, our challenge reduces to finding the values of $\left[x_{1},y_{1},x_{2},y_{2},z_{1}\right]all\in \left[0,1\right]$ that maximize Equations (3) and (4):

(3) $$\begin{array}{cc} P(B=|0\rangle ) & =f_{I}\left(x_{1},y_{1},x_{2},y_{2},z_{1}\right)=\left|\right|a_{0}b_{0}c_{0}{\left|\right|}^{2}+\left|\right|a_{0}b_{0}c_{1}{\left|\right|}^{2}+\left|\right|a_{1}b_{0}c_{0}{\left|\right|}^{2}+\left|\right|a_{1}b_{0}c_{1}{\left|\right|}^{2} \end{array}$$

(4) $$\begin{array}{cc} P(C=|0\rangle ) & =g_{I}\left(x_{1},y_{1},x_{2},y_{2},z_{1}\right)=\left|\right|a_{0}b_{0}c_{0}{\left|\right|}^{2}+\left|\right|a_{0}b_{1}c_{0}{\left|\right|}^{2}+\left|\right|a_{1}b_{0}c_{0}{\left|\right|}^{2}+\left|\right|a_{1}b_{1}c_{0}{\left|\right|}^{2}. \end{array}$$

Based on the principles of quantum circuit design exposed in [19], we present the quantum circuit that represents the interactions of the decision network exposed in Figure 1I expressed by Equation (5):
(5)
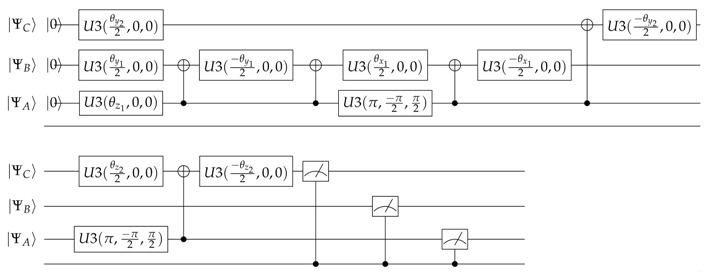


This circuit presents three qubits $|\Psi_{A}\rangle$, $|\Psi_{B}\rangle$, and $|\Psi_{C}\rangle$ which are rotated through quantum operators. The respective interpretation of these rotations and the equations to calculate them are described in Table 1.

### 2.2. Quantum Circuit–Case II–Agents B and C Have Communication between Each Other

In this case, as shown in Figure 1II, we will represent a three-qubit system. As explained in [19,21], this requires the use of an additional ancilla-qubit $q^{*}$, whose state is given by $|\Psi^{*}\rangle$, that will allow us to use certain quantum operations that would otherwise be unfeasible. As a consequence, we are faced with a four qubit system whose aggregate state can be expressed as the tensorial product of the individual qubits. The multiple *qubit* state can be expressed as a linear combination of the |0〉 and |1〉 states, then the aggregated state can be represented as in Equation (6).

(6) $$\begin{array}{cc} |\Psi \rangle & =|\Psi_{A}\rangle ⊗|\Psi_{B}\rangle ⊗|\Psi_{C}\rangle ⊗|\Psi^{*}\rangle \\ \; & =a_{0}b_{0}c_{0}d_{0}|0000\rangle +a_{0}b_{0}c_{0}d_{1}|0001\rangle +a_{0}b_{0}c_{1}d_{0}|0010\rangle +a_{0}b_{0}c_{1}d_{1}|0011\rangle \\ \; & +a_{0}b_{1}c_{0}d_{0}|0100\rangle +a_{0}b_{1}c_{0}d_{1}|0101\rangle +a_{0}b_{1}c_{1}d_{0}|0110\rangle +a_{0}b_{1}c_{1}d_{1}|0111\rangle \\ \; & +a_{1}b_{0}c_{0}d_{0}|1000\rangle +a_{1}b_{0}c_{0}d_{1}|1001\rangle +a_{1}b_{0}c_{1}d_{0}|1010\rangle +a_{1}b_{0}c_{1}d_{1}|1011\rangle \\ \; & +a_{1}b_{1}c_{0}d_{0}|1100\rangle +a_{1}b_{1}c_{0}d_{1}|1101\rangle +a_{1}b_{1}c_{1}d_{0}|1110\rangle +a_{1}b_{1}c_{1}d_{1}|1111\rangle \end{array}$$

where:
$|\Psi_{A}\rangle =a_{0}|0\rangle +a_{1}|1\rangle$$\;a_{i}\in C^{2}$$|\Psi_{B}\rangle =b_{0}|0\rangle +b_{1}|1\rangle$$\;b_{i}\in C^{2}$$|\Psi_{C}\rangle =c_{0}|0\rangle +c_{1}|1\rangle$$\;c_{i}\in C^{2}$$|\Psi^{*}\rangle =d_{0}|0\rangle +d_{1}|1\rangle$$\;d_{i}\in C^{2}$.

Thus it can be said that the quantum system of 4 qubits can be described by a $2^{4}$-dimensional complex unit vector $|\Psi \rangle \in C^{2}$.

To describe the case in which agents *B* and *C* have communication between each other we need following parameters:

- $1-z_{11}=P\left(A=|0\rangle \right)=1-P\left(A=|1\rangle \right)$. Probability of alignment of node *A*.
- $z_{21}=P\left(B=|1\rangle |A=|1\rangle \right)$. Probability of no–alignment of node *B* conditioned to the state of no–alignment of node *A*.
- $z_{22}=P\left(B=|1\rangle |A=|0\rangle \right)$. Probability of no–alignment of node *B* conditioned to the state of alignment of node *A*.
- $x_{11}=P\left(C=|1\rangle |A,B=|11\rangle \right)$. Probability of no–alignment of node *C* conditioned to the state |11〉 of the waveform $|\Psi_{A}\rangle ⊗|\Psi_{B}\rangle$.
- $y_{11}=P\left(C=|1\rangle |A,B=|10\rangle \right)$. Probability of no–alignment of node *C* conditioned to the state |10〉 of the waveform $|\Psi_{A}\rangle ⊗|\Psi_{B}\rangle$.
- $x_{21}=P\left(C=|1\rangle |A,B=|00\rangle \right)$. Probability of no–alignment of node *C* conditioned to the state |00〉 of the waveform $|\Psi_{A}\rangle ⊗|\Psi_{B}\rangle$.
- $y_{21}=P\left(C=|1\rangle |A,B=|01\rangle \right)$. Probability of no–alignment of node *C* conditioned to the state |01〉 of the waveform $|\Psi_{A}\rangle ⊗|\Psi_{B}\rangle$.

Mathematically speaking, we intend to find the values of $\left(x_{11},y_{11},x_{21},y_{21},z_{11},z_{21},z_{22}\right)$ that maximize the functions $P\left(B=|0\rangle \right)=f_{II}\left(x_{11},y_{11},x_{21},y_{21},z_{11},z_{21},z_{22}\right)$ and $P\left(C=|0\rangle \right)=g_{II}\left(x_{11},y_{11},x_{21},y_{21},z_{11},z_{21},z_{22}\right)$. In other words, our challenge reduces to finding the values of $\left[x_{11},y_{11},x_{21},y_{21},z_{11},z_{21},z_{22}\right]all\in \left[0,1\right]$ that maximize Equations (7) and (8):

(7) $$\begin{array}{cc} P(B=|0\rangle ) & =f_{II}\left(x_{11},y_{11},x_{21},y_{21},z_{11}\right)\\ \; & =\left|\right|a_{0}b_{0}c_{0}d_{0}{\left|\right|}^{2}+\left|\right|a_{0}b_{0}c_{0}d_{1}{\left|\right|}^{2}\\ \; & +\left|\right|a_{0}b_{0}c_{1}d_{0}{\left|\right|}^{2}+\left|\right|a_{1}b_{0}c_{0}d_{0}{\left|\right|}^{2}\\ \; & +\left|\right|a_{0}b_{0}c_{1}d_{1}{\left|\right|}^{2}+\left|\right|a_{1}b_{0}c_{0}d_{1}{\left|\right|}^{2}\\ \; & +\left|\right|a_{1}b_{0}c_{1}d_{0}{\left|\right|}^{2}+\left|\right|a_{1}b_{0}c_{1}d_{1}{\left|\right|}^{2} \end{array}$$

(8) $$\begin{array}{cc} P(C=|0\rangle ) & =g_{II}\left(x_{11},y_{11},x_{21},y_{21},z_{11}\right)\\ \; & =\left|\right|a_{0}b_{0}c_{0}d_{0}{\left|\right|}^{2}+\left|\right|a_{0}b_{0}c_{0}d_{1}{\left|\right|}^{2}\\ \; & +\left|\right|a_{0}b_{1}c_{0}d_{0}{\left|\right|}^{2}+\left|\right|a_{1}b_{0}c_{0}d_{0}{\left|\right|}^{2}\\ \; & =\left|\right|a_{1}b_{1}c_{0}d_{0}{\left|\right|}^{2}+\left|\right|a_{1}b_{0}c_{0}d_{1}{\left|\right|}^{2}\\ \; & =\left|\right|a_{0}b_{1}c_{0}d_{1}{\left|\right|}^{2}+\left|\right|a_{1}b_{1}c_{0}d_{1}{\left|\right|}^{2}. \end{array}$$

Based on the principles of quantum circuit design exposed in [19], we present the quantum circuit that represents the interactions of the decision network exposed in Figure 1II expressed by Equation (9):
(9)
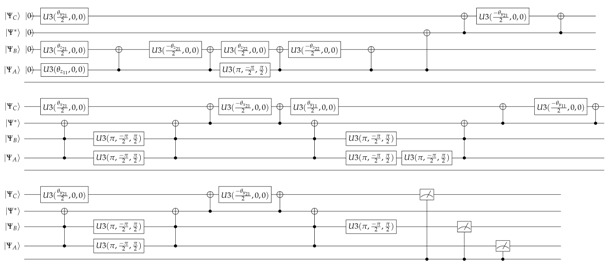


This circuit presents four qubits $|\Psi_{A}\rangle$, $|\Psi_{B}\rangle$, $|\Psi_{C}\rangle$, and $|\Psi^{*}\rangle$ which are rotated through quantum operators. The respective interpretation of these rotations and the equations to calculate them are described in Table 2.

## 3. Case Study

In the following case study we move on to simulate thousands of configurations of the parameters presented in the quantum circuit Equations (5) and (9) to understand those that provide a maximization of the alignment probabilities of agents *B* and *C*, P(B=|0〉), and P(C=|0〉), given by Equations (7) and (8) respectively. The circuits were simulated on a *qiskit* tool, a Python-based [33] quantum computing platform developed by IBM [34], and the code and additional results can be accessed in this Open Access Repository. We will evaluate these results in Section 4.

### 3.1. Simulation–Case I–Agents B and C Have No Communication between Each Other

As shown in Equation (5), in the case of agents *B* and *C* with no communication between each other, we find five variables. As a consequence of Equation (2), the sample space is too large to use brute force to explore the phase space associated with the solutions and therefore we will proceed to set one or more variables and see how the others behave by means of exploratory graphs.

First of all we investigate the relationship between the alignment of agents *B* and *C* when the alignment of *A* changes.

**Figure 2:**
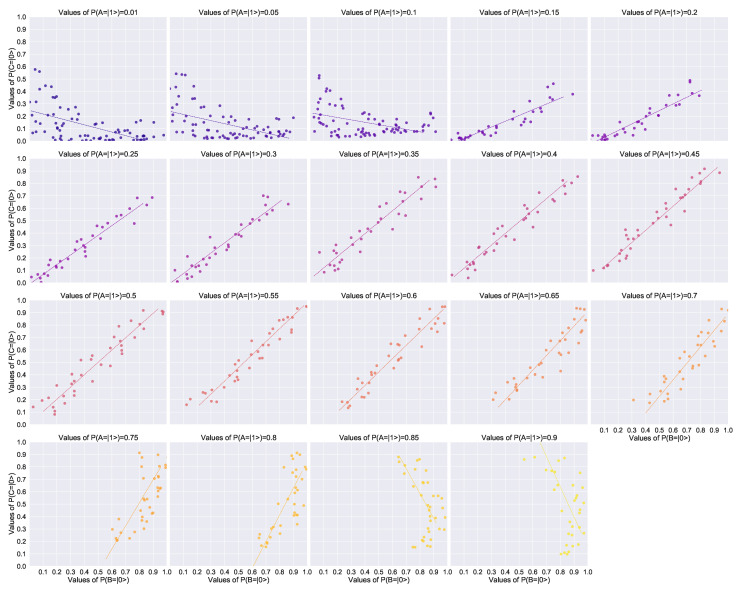
Correlation between P(B=|0〉) and P(C=|0〉) for different values of $z_{1}=P\left(A=|1\rangle \right)\in \xi_{1}$ for the case of no communication between *B* and *C*.

In Figure 2 we show the results of the simulations obtained by representing the alignment probabilities of agents *B* and *C*, P(B=|0〉), and P(C=|0〉), for each value of $z_{1}=P\left(A=|1\rangle \right)\in \xi_{1}$ and all possible combinations of $\left\{x_{1},y_{1},x_{2},y_{2}\right\}\in \xi_{1}$, whereas $\xi_{1}=\left\{0.1,0.2,0.3,0.4,0.5,0.6,0.7,0.8,0.9\right\}$.

Next we show how the alignment of agents *B* and *C* changes when the alignment probability of *A* changes, when the relative probability of not–alignment of *B* and *C* conditioned to the not–alignment state of *A* are the same.

**Figure 3:**
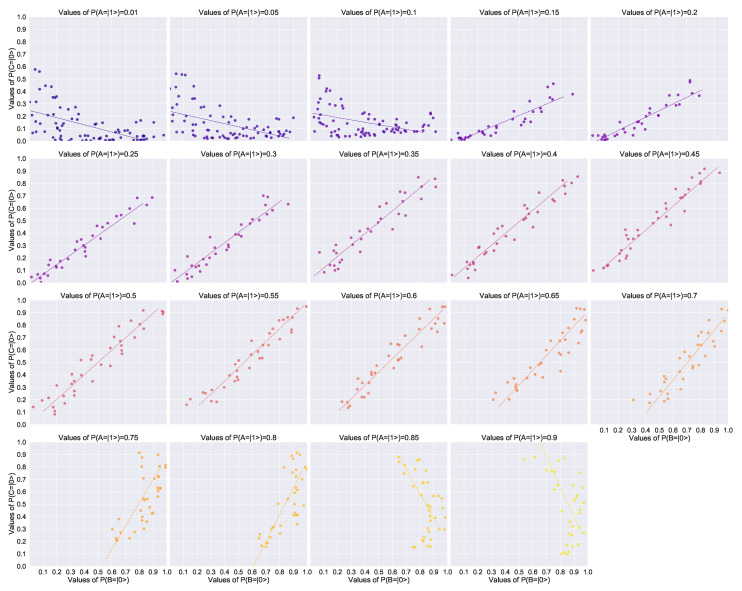
Correlation between P(B=|0〉) and P(C=|0〉) for different values of $y_{1}=P\left(B=|1\rangle |A=|1\rangle \right)=y_{2}=P\left(C=|1\rangle |A=|1\rangle \right)\in \xi_{2}$ for the case of no communication between *B* and *C*.

In Figure 3 we show the results of the simulations obtained by representing the alignment probabilities of agents *B* and *C*, P(B=|0〉) and P(C=|0〉), for each value of $y_{1}=P\left(B=|1\rangle |A=|1\rangle \right)=y_{2}=P\left(C=|1\rangle |A=|1\rangle \right)\in \xi_{2}$, and all possible combinations of $\left\{z_{1},x_{1},x_{2}\right\}\in \xi_{2}$, whereas $\xi_{2}=\left\{0.01,0.05,0.1,0.2,0.3,0.4,0.5,0.6,0.7,0.8,0.9\right\}$.

**Figure 4:**
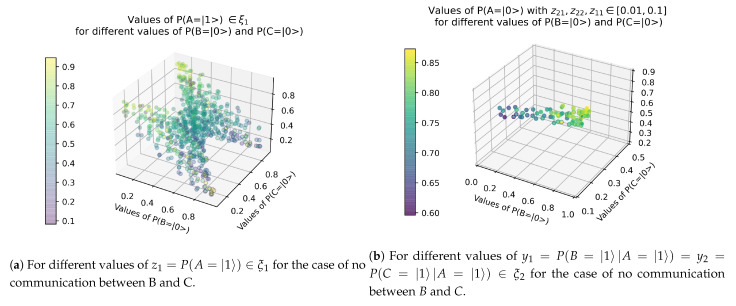
Correlation between P(B=|0〉) and P(C=|0〉).

In order to compare these results with those of Section 3.2. The results of Figure 2 and Figure 3 are summarized in 3D in Figure 4a,b respectively.

### 3.2. Simulation–Case II–Agents B and C Have Communication between Each Other

As shown in Equation (9), in the case of agents *B* and *C* with communication between each other, we find seven variables. As a consequence of Equation (6), the sample space is too large to use brute force to explore the phase space associated with the solutions.

As in the previous case, we intend to investigate the behavior of the alignment probabilities of agents *B* and *C*, P(B=|0〉) and P(C=|0〉). The results obtained in [20] indicate that the alignment probability of P(A=|0〉) that allows for an alignment of the higher nodes is greater or equal than 90%.

Therefore we set the value of $z_{11}=P\left(A=|1\rangle \right)\in \left[0.01,0.1\right]$, and vary accordingly the values of $z_{21}=P\left(B=|1\rangle \right|A=|1\rangle =z_{22}=P\left(B=|1\rangle |A=|0\rangle \right)both\in \left[0.01,0.9\right]$, with changing values of $x_{11}=x_{21}=x_{21}=y_{21}$, to observe the change in the alignment probabilities of agents *B* and *C*, P(B=|0〉) and P(C=|0〉).

**Figure 5:**
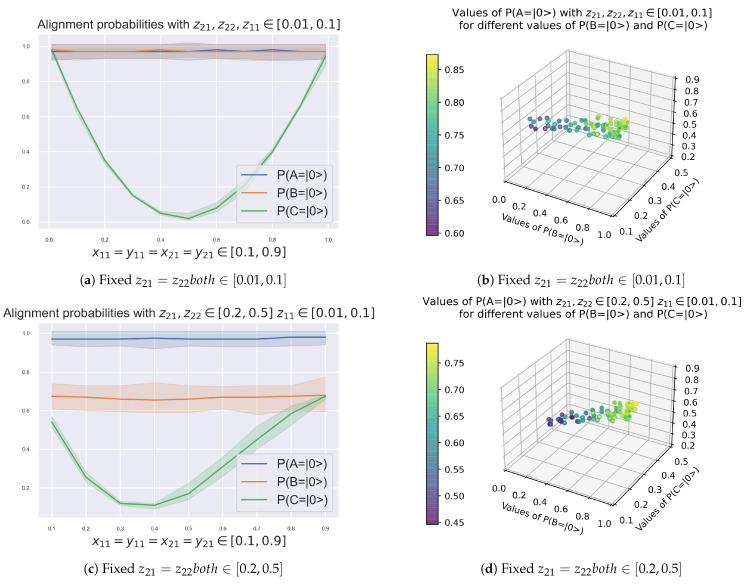

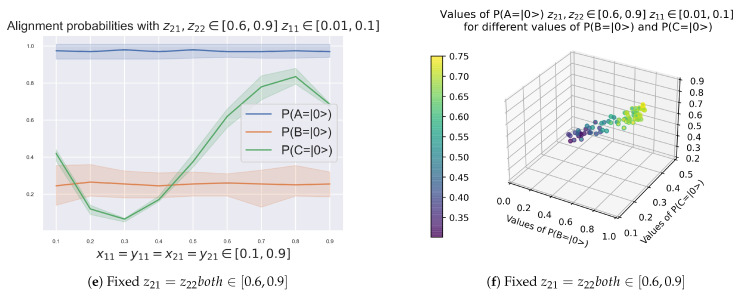
Alignment Probabilities of P(A=|0〉),P(B=|0〉) and P(C=|0〉) with $z_{11}=P\left(A=|1\rangle \right)\in \left[0.01,0.1\right]$ for different values of fixed $z_{21}=P\left(B=|1\rangle |A=|1\rangle \right)=z_{22}=P\left(B=|1\rangle |A=|0\rangle \right)$, and combinations of $x_{11}=x_{21}=x_{21}=y_{21}$.

This is shown in Figure 5:

- Figure 5a, and its equivalent in 3D Figure 5b, for P(B=|0〉) and P(C=|0〉) with fixed $z_{21}=P\left(B=|1\rangle \right|A=|1\rangle =z_{22}=P\left(B=|1\rangle |A=|0\rangle \right)both\in \left[0.01,0.1\right]$,
- Figure 5c, and its equivalent in 3D Figure 5d, for P(B=|0〉) and P(C=|0〉) with fixed $z_{21}=P\left(B=|1\rangle \right|A=|1\rangle =z_{22}=P\left(B=|1\rangle |A=|0\rangle \right)both\in \left[0.2,0.5\right]$, and
- Figure 5e, and its equivalent in 3D Figure 5f, for P(B=|0〉) and P(C=|0〉) with fixed $z_{21}=P\left(B=|1\rangle \right|A=|1\rangle =z_{22}=P\left(B=|1\rangle |A=|0\rangle \right)both\in \left[0.6,0.9\right]$.

## 4. Discussion

In Section 4 we proceed to discuss the results **R** obtained from the simulations.

### 4.1. Discussion–Case I–Agents B and C Have No Communication between Each Other

In the case in which agents *B* and *C* have no communication between each other, we can derive following results:

- **R1**. Agents *B* and *C* have an antagonistic alignment probability. The two never have a high probability of alignment simultaneously. In Figure 2 we can see how, for both high and low values of alignment for node *A*, P(A=|0〉)=0.9 or P(A=|0〉)=0.1 respectively, the alignment probabilities of agents *B* and *C* have a negative correlation. When one of the two has high alignment probabilities, the other has low ones.
- **R2**. Agents *B* and *C* only agree by chance. In Figure 2 we can see how, as agent *A* approaches its random alignment probability of 50%, the alignment probabilities of *B* and *C* become homogeneous until reaching the 50% value as well.
- **R3**. Quantum phase transition with 90% alignment probability of node *A*. The representations of Figure 3 are particular cases of the general solution of Figure 2. In both we can observe a sharp change of slope of the regression between the alignment probabilities of *B* and *C*. This clearly indicates a quantum phase change at the point where the probability of non–alignment of agent *A* is 10%, P(A=|1〉)=0.1. In more detail, the observed results show:
  - As shown in Figure 3, if the alignment probability of *A* is very high, P(A=|0〉)>0.9 (or P(A=|1〉)<0.1), and the probability that *B* and *C* are in non–alignment, provided that *A* is in non–alignment, are equal, $y_{1}=P\left(B=|1\rangle |A=|1\rangle \right)=y_{2}=P\left(C=|1\rangle |A=|1\rangle \right)$, then the alignment probability of *C* is very low and does not vary with the alignment probability of *B*;
  - As shown in Figure 3, if the alignment probability of *A* is not high, 0.15<P(A=|1〉)<0.90, and the probability that *B* and *C* are in non–alignment, provided that *A* is in non–alignment, are equal, $y_{1}=P\left(B=|1\rangle |A=|1\rangle \right)=y_{2}=P\left(C=|1\rangle |A=|1\rangle \right)$, then the alignment probability of *B* and *C* present a positive correlation.

### 4.2. Discussion–Case II–Agents B and C Have Communication between Each Other

In the case in which agents *B* and *C* have communication between each other, we can derive following results:

- **R4**. When *B* and *C* are entangled, they work as one. As shown in Figure 5a, when $x_{11}=x_{21}=x_{21}=y_{21}=z_{21}=z_{22}all\in ]0,0.1]\cup [0.9,1[$, the quantum circuit is identical to that of one qubit reporting to other qubit shown in [20], and behaves in a similar manner.
- **R5**. Agents *B* and *C* interchange energy. Lowering the probability of alignment of node *B*, P(B=|0〉), which can be understood as its energy, while maintaining P(A=|1〉)∈[0.01,0.1], shows how P(C=|0〉) behaves with changing $x_{11}=x_{21}=x_{21}=y_{21}=z_{21}=z_{22}$. The curves shown quantify this interaction.

## 5. Conclusions, Limitations and Further Steps

### 5.1. Conclusions–Case I–Agents B and C Have No Communication between Each Other

**M1**. The management conclusion derived from **R1** for subordinate agent *A* is staggering and somehow tragic: If the two bosses do not communicate with each other, *A* will never be able to serve them in such a way that both are simultaneously in alignment. It does not matter what *A* does. This could lead one to believe that agent *A*’s motivation to provide a contribution to the value chain may be diminished due to the very organizational structure in which they are immersed, regardless of capabilities, skills, or attitudes. The organizational design would therefore impose undesirable boundary conditions for the adequate development of the activity of the subordinate node.

**M2**. The conclusions derived from the **R2** result are not very encouraging for management either. In case the two superior agents do not communicate between them, their joint alignment is always around the point of equilibrium, which is the probability given by the chance. As long as the subordinate node has a higher or lower probability of alignment, their positions will be more or less differentiated. This would imply that the node would tend not to position itself with either of the two nodes to which it reports and the expected behavior on its part would be one of a lack of decision-making that could potentially jeopardize the efficiency of the associated value creation processes.

**M3**. The conclusions derived from the **R3** confirm the results obtained in [20]: Only a strong alignment probability at lower reporting levels enables alignment at higher levels. It seems that empirically the threshold is set by 90%. To grow the organizational network towards strategic objectives, it is necessary to ensure asymptotic stability at the operational levels of the organization. These lower levels are generally the levels closest to the creation of value and it seems logical that they are the sustaining base of the organizational structure.

### 5.2. Conclusions–Case II–Agents B and C Have Communication between Each Other

**M4**. The conclusions derived from the **R4** result is that high levels of alignment in both reporting agents *A* and *B* do not imply a high level of alignment of node *C*. In the case in which *B* and *C* high levels of alignment in node *C* are only attained for an entangled system in which *A*, *B*, and *C* are highly dependent, given the condition $x_{11}=x_{21}=x_{21}=y_{21}=z_{21}=z_{22}all\in ]0,0.1]\cup [0.9,1[$.

**M5**. The conclusions derived from **R5** show that the interaction between the superior agents *B* and *C* becomes manifest when the alignment probability of *A* is fixed at values higher than 90%. Both superior agents *B* and *C* present a non-linear interaction, and depending on what agent should be prioritized, strategies can be then taken towards one or toward other.

The proposed approach can be useful to provide knowledge in different areas, such as project management. For example, this work shows a situation where project specialists are responsible to two managers. Case I would reflect projects that operate between the boundaries of divisions with poor communication and inefficient information exchange. On the other hand, Case II would simulate projects operating in a more participating environment. In summary, QSOD could help to simulate a number of different organization designs to better understand how they impact on the achievement of the project objectives and to orientate the project organization accordingly. Thus, it could be used as a project management tool since it enables the assessment of the project development in the context of several different scenarios.

This study’s major shortcoming is that it deals solely with two of all possible configurations involving three agents. Furthermore, quantum circuit simulations were made in a classical computer simulator. This reduces certainly their statistical significance, however, this circumstance is irrelevant for our study at this time and can be neglected.

The results obtained studying the QSOD case of 3 qubits, in which one reports to two, and leads to new and challenging research questions. To continue providing valuable contributions to Industry 4.0 leaders as well as members of the research community in general, future efforts on this research line will concentrate on studying the behavior of more complex QSOD configurations. For instance, the strategic design of organizations in Industry 4.0 environments is reinforced with the new knowledge derived from the analyses and results obtained in this work, since these allow a better understanding of basic motives that will later be added to explain classic organizational structures such as the matrix form. To do this, these results must be extended to configurations with 4 qubits.

## Figures and Tables

**Table 1:** Qubit angles of rotation.

Qubit	Interpretation	Equation
$|\Psi_{A}\rangle$	The probability $z_{1}=P\left(A=|1\rangle \right)$ of qubit $|\Psi_{A}\rangle$ to be in not–alignment translates into the rotation angle $\theta_{z_{1}}$.	$\theta_{z_{1}}=2\arctan\sqrt{\frac{z_{1}}{1-z_{1}}}$
$|\Psi_{B}\rangle$	The conditional probability $x_{1}=P\left(B=|1\rangle |A=|0\rangle \right)$ of qubit $|\Psi_{B}\rangle$ to be in not–alignment depending on the probability of $|\Psi_{A}\rangle$ to be in the state |0〉 translates into rotation angle $\theta_{x_{1}}$.	$\theta_{x_{1}}=2\arctan\sqrt{\frac{x_{1}}{1-x_{1}}}$
	The conditional probability $y_{1}=P\left(B=|1\rangle |A=|1\rangle \right)$ of qubit $|\Psi_{B}\rangle$ to be in not–alignment depending on the probability of $|\Psi_{A}\rangle$ to be in the state |1〉 translates into rotation angle $\theta_{y_{1}}$.	$\theta_{y_{1}}=2\arctan\sqrt{\frac{y_{1}}{1-y_{1}}}$
$|\Psi_{C}\rangle$	The conditional probability $x_{2}=P\left(C=|1\rangle |A=|0\rangle \right)$ of qubit $|\Psi_{C}\rangle$ to be in not–alignment depending on the probability of $|\Psi_{A}\rangle$ to be in the state |0〉 translates into rotation angle $\theta_{x_{2}}$.	$\theta_{x_{2}}=2\arctan\sqrt{\frac{x_{2}}{1-x_{2}}}$
	The conditional probability $y_{2}=P\left(C=|1\rangle |A=|1\rangle \right)$ of qubit $|\Psi_{C}\rangle$ to be in not–alignment depending on the probability of $|\Psi_{A}\rangle$ to be in the state |1〉 translates into rotation angle $\theta_{y_{2}}$.	$\theta_{y_{2}}=2\arctan\sqrt{\frac{y_{2}}{1-y_{2}}}$

**Table 2:** Qubit angles of rotation.

Qubit	Interpretation	Equation
$|\Psi_{A}\rangle$	The probability $z_{11}=P\left(A=|1\rangle \right)$ of qubit $|\Psi_{A}\rangle$ to be in not–alignment translates into the rotation angle $\theta_{z_{11}}$.	$\theta_{z_{11}}=2\arctan\sqrt{\frac{z_{11}}{1-z_{11}}}$
$|\Psi_{B}\rangle$	The conditional probability $z_{21}=P\left(B=|1\rangle |A=|1\rangle \right)$ of qubit $|\Psi_{B}\rangle$ to be in not–alignment depending on the probability of $|\Psi_{A}\rangle$ to be in the state |1〉 translates into rotation angle $\theta_{z_{21}}$.	$\theta_{z_{21}}=2\arctan\sqrt{\frac{z_{21}}{1-z_{21}}}$
	The conditional probability $z_{22}=P\left(B=|1\rangle |A=|0\rangle \right)$ of qubit $|\Psi_{B}\rangle$ to be in not–alignment depending on the probability of $|\Psi_{A}\rangle$ to be in the state |0〉 translates into rotation angle $\theta_{z_{22}}$.	$\theta_{z_{22}}=2\arctan\sqrt{\frac{z_{22}}{1-z_{22}}}$
$|\Psi_{C}\rangle$	The conditional probability $x_{11}=P\left(C=|1\rangle |A,B=|11\rangle \right)$ of qubit $|\Psi_{C}\rangle$ to be in not–alignment depending on the probability of the waveform $|\Psi_{A}\rangle ⊗|\Psi_{B}\rangle$ to be in the state |11〉 translates into rotation angle $\theta_{x_{1}1}$.	$\theta_{x_{11}}=2\arctan\sqrt{\frac{x_{11}}{1-x_{11}}}$
	The conditional probability $y_{11}=P\left(C=|1\rangle |A,B=|10\rangle \right)$ of qubit $|\Psi_{C}\rangle$ to be in not–alignment depending on the probability of the waveform $|\Psi_{A}\rangle ⊗|\Psi_{B}\rangle$ to be in the state |10〉 translates into rotation angle $\theta_{y_{11}}$.	$\theta_{y_{11}}=2\arctan\sqrt{\frac{y_{11}}{1-y_{11}}}$
	The conditional probability $x_{21}=P\left(C=|1\rangle |A,B=|00\rangle \right)$ of qubit $|\Psi_{C}\rangle$ to be in not–alignment depending on the probability of the waveform $|\Psi_{A}\rangle ⊗|\Psi_{B}\rangle$ to be in the state |00〉 translates into rotation angle $\theta_{x_{2}1}$.	$\theta_{x_{21}}=2\arctan\sqrt{\frac{x_{21}}{1-x_{21}}}$
	The conditional probability $y_{21}=P\left(C=|1\rangle |A,B=|01\rangle \right)$ of qubit $|\Psi_{C}\rangle$ to be in not–alignment depending on the probability of the waveform $|\Psi_{A}\rangle ⊗|\Psi_{B}\rangle$ to be in the state |01〉 translates into rotation angle $\theta_{y_{21}}$.	$\theta_{y_{21}}=2\arctan\sqrt{\frac{y_{21}}{1-y_{21}}}$
$|\Psi^{*}\rangle$	The ancilla qubit $|\Psi^{*}\rangle$ is a support qubit and as such is not subject to any conditional probability rotation.	

## Abbreviations

The following abbreviations are used in this manuscript:

QSOD	Quantum Strategic Organizational Design
KPI	Key Performance Indicators
